# Actions and perceived impact of African swine fever control measures along the smallholder pig value chain in Uganda

**DOI:** 10.1007/s11250-023-03828-5

**Published:** 2023-11-21

**Authors:** Tonny Aliro, Walter Odongo, Karl Ståhl, Michel Mainack Dione, Daniel Micheal Okello, Charles Masembe, Erika Chenais

**Affiliations:** 1 Faculty of Agriculture and Environment, Gulu University, P. O. Box 166, Gulu, Uganda; 2 Department of Disease Control and Epidemiology, National Veterinary Institute, Uppsala, Sweden; 3 International Livestock Research Institute (ILRI), P. O. Box 30709, Nairobi, Kenya; 4 College of Natural Sciences, Makerere University, P.O. Box 7062, Kampala, Uganda

**Keywords:** ASF quarantine, Control, Economic impact, Smallholder pig value chain, Stakeholders, Uganda

## Abstract

Pig production in Uganda is constrained by African swine fever (ASF) which is endemic in the country. Current measures taken by the Government of Uganda in controlling ASF outbreaks include trade and livestock movement restrictions, called “quarantine.” Little is known about the actions of, and impact of value chain actors in response to ASF quarantines. This study describes actions that different stakeholders in the smallholder pig value chain took, and the perceived economic impact, during ASF quarantines. Data was collected in ten focus group discussions (FGD) using participatory epidemiology tools and two key informants’ (KIs) interviews with District Veterinary Officers (DVOs) of Kisoro and Moyo districts in Uganda. The results show that during ASF quarantine, pig value chain actors shifted their activities from formal places such as livestock markets, slaughter slabs, pork butcheries and pork joints to informal places such as farmers’ homesteads. Farmers were perceived the most economically affected stakeholder group with forgone income due to unsold pigs, costs for implementing biosecurity measures and extra costs for feeding unsold pigs being the major perceived causes of the losses. The continued trade in pigs and pig products in informal marketplaces suggests that quarantine might not be effective for hindering activities that might spread ASF in these settings. The perceived economic losses provide an insight into the negative economic impact of the quarantine for the different stakeholders.

**Supplementary Information:**

The online version contains supplementary material available at 10.1007/s11250-023-03828-5.

## Introduction

Pig production in Uganda, mainly represented by subsistence smallholders farming with free-range management, has gained prominence (Tatwangire 2014). This has led to an increase in pig numbers from 3.5 million pigs in 2014 to 4.47 million pigs in 2018 (UBOS 2018). The pig sector is, however, affected by several constraints that hinder its further development, including the endemic occurrence of African swine fever (ASF). ASF is a viral disease affecting domestic pigs and wild boar with a serious, haemorrhagic fever that in most cases leads to death within one week after infection (Penrith et al. 2013). The sylvatic epidemiological cycle of ASF is present in Uganda, but as in most parts of the world disease spread is mainly occurring in the domestic pig epidemiological cycle (Penrith et al. 2019). ASF spread in the domestic pig cycle involves direct and indirect contact between naïve and infected pigs and pork products, and is driven by human activities along the value chain such as slaughter and trade in live pigs and pork products. Transmission is exacerbated by low levels of biosecurity and the free range type of management that is common in most smallholder pig farming systems in Uganda (Chenais et al. 2017a).

Despite recent progress in ASF vaccine development, prevention and control of ASF is largely through biosecurity and outbreak management (Penrith et al. 2019; Dixon et al. 2020). In Uganda, current ASF control measures include passive surveillance and trade and livestock movement restrictions, called quarantine, upon confirmation of outbreaks (Wesonga et al. 2018). While previous studies in Uganda have described ASF epidemiology, virology, biosecurity implementation and ASF impact at farm level (Chenais 2017; Chenais et al. 2017a, 2019; Masembe et al. 2018; Ouma et al. 2018) less is known about the implementation of ASF control measures during outbreaks, as well as the impact of these control measures on the different stakeholders along the value chain. To improve ASF control, it is important to understand what stakeholders actually do during quarantine, and how not only the disease but also the control of the disease impact stakeholders. This study aimed at assessing actions taken by different stakeholders and the perceived economic impact during quarantine.

## Material and methods

### Study area

**Fig. 1:**
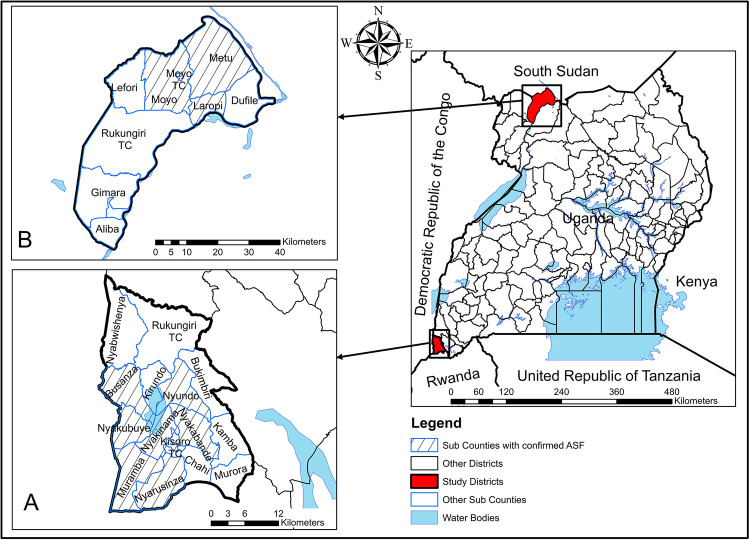
Maps of Kisoro (**A**) and Moyo (**B**) districts showing the sub-counties indicated red where data were collected. These maps were created using ArcGIS version 10.8.0

This study was conducted in 2019 in two districts, Moyo and Kisoro (Fig. 1) in Uganda. Between 2015 and 2018, the veterinary authority in Uganda, Commissioner of Animal Health, imposed quarantine during confirmed outbreaks of ASF in two districts in Uganda. Restrictions were effective in Kisoro district from July 2015 to January 2016 and in Moyo district from March to November 2018. These two districts were selected since quarantine was imposed at all nodes along the pig value chain in all sub-counties in these districts. Based on confirmed ASF cases, eight sub-counties were selected in Kisoro, and four in Moyo.

### Data collection

The study applied participatory epidemiology tools in focus group discussions (FGDs) and key informants’ interviews (KIIs). The field research team was composed of the first author (TA) together with one facilitator and one notetaker for each district. The facilitator and the notetaker pair were both proficient in both English and local language (Rufumbira in Kisoro and Madi in Moyo). Both the facilitators and the notetakers had educational background in livestock production and management and were trained in qualitative and quantitative research approaches and the procedure for data collection prior to data collection. All interviews started with taking participants through the purpose of the study, explaining that participation was voluntary, and assuring confidentiality. Participants signed a written consent form to these effects.

### Key informant interviews (KIIs)

The District Veterinary Officers (DVOs) of the study districts were selected as key informants (KIs) based on their knowledge of guidelines and policies on animal disease control, and for being in charge of livestock disease management (surveillance, control and reporting) in their respective districts. The first author interviewed key informants (in English) before the first FGD was conducted in each district. This interview was done with the purpose of informing the FGD topic guides. The KIIs followed a checklist of topics regarding marketing of pig and pig products and sourcing of pig breeding stock (See Annex 01). The key informants were additionally asked to describe the local pig value chain, including pig market linkages and processes from producers to final pork consumers in their districts at the time of restrictions. Notes were taken on paper.

### Focus group discussions (FGDs)

In each district, focus groups were constructed to be homogenous regarding the participants’ role in the value chain (farmers, traders and veterinarians respectively), but to include both men and women in each group (see Tables 1 and 2). Two FGDs with farmers (hereafter called farmer FGDs and with group identity (ID) FGD1, 2, 6 and 7), two FGDs with other stakeholders in the value chain such as middlemen, butchers (fresh pork retailers) and pork-joint^1^ owners (roasted pork retailers) (hereafter called trader FGDs and with group ID FGD3, 4, 8 and 9), and one FGD with veterinarians (hereafter called veterinarian FGDs and with group ID FGD 5 and 10) were held in each district. The groups included between four and 14 participants, a group size that was deemed optimal for active participation in the discussions (Krueger & Casey, 2000). Farmers were selected for the FGDs using predetermined inclusion criterion: that the farmer was a resident in a subcounty that had confirmed ASF during the restriction period, had pigs during the period of restriction, and that his/her pig herd was not affected during the restriction period. In addition to farmers, traders were selected for the FGDs using the following inclusion criterium: that the trader operated pig or pork business in one of the sub-counties that had confirmed ASF at the time of restrictions. Veterinarians who worked as livestock extension workers in the affected sub-counties during the restriction period were further selected for inclusion.

**Table 1:** Composition of participants in FGDs from a study performed in Moyo and Kisoro districts of Uganda in 2019. *FGDs* focus group discussions; *PJO* pork joint owner

District	Categories of participants in FGDs						Total
	Farmers	Butchers	Butchers & PJO	PJO	Middlemen	Veterinarians	
Moyo	22	0	6	1	2	5	36
Kisoro	27	1	4	2	3	4	41
Total	49	1	10	3	5	9	77

**Table 2:** Characteristics of participants in an interview study performed in Uganda in 2019

District	Group identity	Male	Female	Total
Moyo	FGD1 (Farmers)	7	3	10
	FGD2 (Farmers)	8	4	12
	FGD3 (Traders)	3	1	4
	FGD4 (Traders)	5	0	5
	FGD5 (Veterinarians)	4	1	5
	KII-1 (DVO)	1	0	1
Kisoro	FGD6 (Farmers)	5	9	14
	FGD7 (Farmers)	6	7	13
	FGD8 (Traders)	5	0	5
	FGD9 (Traders)	3	2	5
	FGD10 (Veterinarians)	3	1	4
	KII-2 (DVO)	1	0	1
Total		51	28	79

The discussion followed a topic guide which included imposed quarantine measures, perceived reasons for imposition, measures taken by actors in response to the restrictions, stakeholders perceived to be economically affected, and ranking of affected stakeholders according to the relative economic impact (Annex 02). Ranking using proportional piling was performed as described by Mariner & Paskin (2000) for the topic of stakeholders that were affected economically. During the FGDs, the facilitator guided the discussion. This included asking participants what they thought about what other participants mentioned, if anyone disagreed or had different opinions to share. Different opinions were discussed and consensus was sought. The FGDs were held in Rufumbira (in Kisoro district) and Madi (in Moyo district) with the facilitator translating to English, sentence by sentence. The first author asked probing questions if something was unclear and took detailed notes. Additional notes were taken by a notetaker. The FGDs lasted on average 80 min. Refreshments in the form of a soft drinks were served during the discussion. Participants were refunded transport expenses at the end of the discussions.

In each FGD, participants were asked to list the restriction measures included in the imposed quarantine. Every restriction measure mentioned by a participant formed the basis of discussion concerning why the participants thought the measure was imposed, and actions taken by the participants in connection to that measure.

Further, the participants identified all stakeholders who could have been affected economically by the quarantine. The facilitator listed these stakeholders on a flip chart sheet placed on the ground. In the next step, one participant was asked to distribute 100 beans between the listed stakeholders to reflect the perceived relative economic impact due to the quarantine. More beans were given to stakeholders perceived as having suffered more economic impact. All participants were encouraged to contribute to the discussion and in the decision for the final distribution of beans. The number of beans allocated to each stakeholder were counted, and the stakeholders ranked according to the number of beans, with the stakeholder receiving the highest number of beans ranked as number 1, and the stakeholder with the second highest number of beans ranked as number 2, and so on for all listed stakeholders. Stakeholders not listed in all the ten FGDs were not considered for analysis. After this, aspects perceived as causing these economic losses were listed for every mentioned stakeholder. This list formed the basis for a subsequent discussion on reasons for the ranking.

### Data analysis

Immediately after the KII in each district, the first author (TA) performed a preliminary analysis of the data by reading through the notes and removing phrases that were repeated, and/or deemed irrelevant to the topic of discussion. After that the KII findings were used to improve the draft FGD topic guide. The notes from the two KIIs were compared to provide information regarding pig marketing from farmers to final consumers, existing local pig value chain, various market linkages and pig breeding that were similar and or different across the two districts.

On each day, at the end of FGDs, the first author and the notetaker compared their field notes, including participants’ details (stakeholder category and gender) to create one final master note for each FGD. Based on these master notes TA and the fifth author (DMO) aggregated data from all the ten FGDs (Tables 1, 2, 3, 4, and 5). Actions taken were recorded and every action mentioned was recorded as mentioned or not mentioned for each FGD. Data referring to economically affected stakeholders ranked by participants in each FGD were merged to give the overall rankings across all ten FGDs. Data referring to perceived economic reasons for ranking stakeholders were recorded as the number of FGDs that mentioned a particular reason under the affected stakeholder. This procedure was done for all the perceived economically affected stakeholders.

**Table 3:**
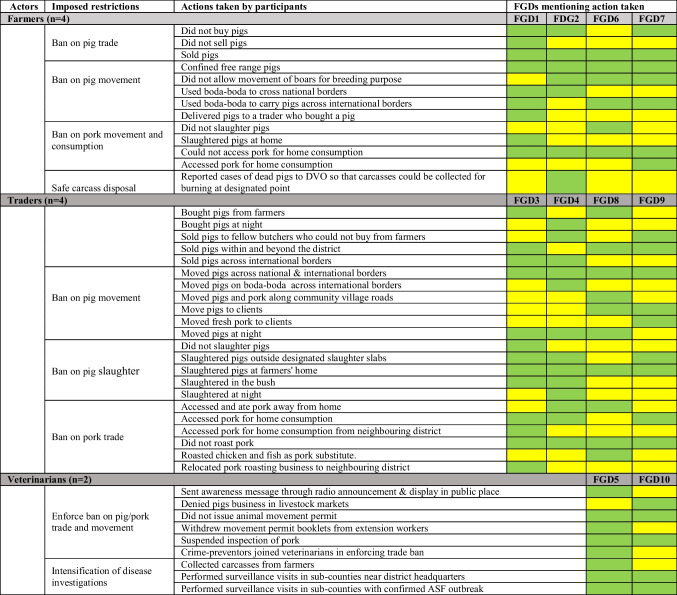
Action taken as reported by stakeholders in response to African swine fever (ASF) quarantine in two districts in Uganda. *FGD* focus group discussion, *DVO* district veterinary officer. Green cells = At least one participant in a FGD mentioned that action; Yellow cells = No participants in a FGD mentioned that action

**Table 4:** Ranking of stakeholders according to the perceived impact of African swine fever quarantine in two districts of Uganda. *FGD* focus group discussion

Group Category	Group identity	Ranking* of the affected stakeholders					
		Farmers	Middlemen	Tax-collectors	Butchers	Consumers	Veterinarians
Farmers	FGD1	1	4	2	3	6	5
	FGD2	1	3	4	2	5	6
	FGD6	1	3	6	2	5	4
	FGD7	2	3	6	1	4	4
Traders	FGD3	3	2	5	1	4	6
	FGD4	1	4	5	2	2	6
	FGD8	2	3	5	1	6	4
	FGD9	1	4	3	2	6	5
Veterinarians	FGD5	1	2	3	3	6	3
	FGD10	1	3	6	2	4	5
Sum of ranking scores		14	31	45	19	48	48
Overall ranking		1	3	4	2	5	5

^*^Ranking 1 = *most affected stakeholder, 5* = *least affected stakeholders*

**Table 5:** Perceived economic losses due to imposed restriction measures on stakeholders *FGD* focus group discussions

Affected stakeholders	Areas of economic impact	Number of FGDs (*n* = 10) that mentioned
Farmers	Forgone revenue for pig sales	10
	Cost incurred for feeding unsold pigs	10
	Cost for biosecurity measures	7
	Cost for veterinary payment	6
	Foregone revenue for boar hire	5
	Cost incurred for feeding confined (instead of free-range) pigs	5
	Foregone revenue for manure sales	4
	Cost for constructing shelter for confining free-range pigs	4
	Cost for pigs’ requirements	4
	Loan servicing	4
	Cost for buying local concoction for treatment of pigs	1
	Cost for waste disposal	1
Butchers and pork joint operators (pork retailers)	Foregone revenue for live pigs and pork sales	10
	Cost for rent of business premises	5
	Cost for relocating business across border	4
	Payment to farmers for pigs bought on credit	4
	Cost for feeding unsold pigs	3
	Unrecovered money for trading license	3
	Foregone revenue for fresh pork that staled in the fridge when the facility was suddenly closed	2
	Loan servicing	2
	Foregone revenue for swill or leftovers sales	1
	Cost for loan for alternative businesses	1
Middlemen	Foregone revenue for pig sales	10
	Loss of revenue as commission payment from butchers	9
	Cost for pigs that died in confinement facility	5
	Loan servicing	5
	Rent for live pig holding facility	3
	Violation fine (when defied the laws)	1
Tax-collectors	Forgone slaughter fee	8
	Foregone market due fee	5
	Foregone market loading fee	5
	Cost for maintaining slaughter slab premises	5
	Foregone release fees collections along highways	3
	Loss of commitment-fee	2
	Cost for paying workers’ salary	2
	Payment for mandatory medical check-up	1
Consumers	Cost for pork substitute	10
	Cost for pork bought illegally	7
	Cost for hiring boda-boda drivers to buy pork across border	6
	Cost for pork bought across border	2
Veterinarians	Foregone pork inspection fee	5
	Foregone movement permit fee	5
	Cost for quarantine enforcement	5
	Loss of pig treatments revenue	4
	Cost for sensitizing the general public	4
	Foregone live pig (market) inspection fee	3
	Cost for carcasses collection	3
	Cost for carcasses disposal	3
	Loss of drugs sales revenue	2
	Cost for sample collection	2

## Results

### KIs and FGD participants

In total, two KIs were interviewed, and 77 participants were included in ten FGDs. The composition of the FGDs varied according to the stakeholders present in each study location. The majority (49/77) of FGD-participants and all key informants were men (see Tables 1 and 2).

### Key informant interviews

According to the respective KIIs, Moyo district did not have any livestock markets at the time of quarantine imposition whereas Kisoro district had weekly livestock markets in two different locations. Unsold pigs would be taken back home by farmers from both livestock markets. Furthermore, pigs bought from livestock markets in Kisoro and at farm-gates in both districts would sometimes be sold in neighbouring districts and countries. Pigs bought for slaughter from livestock markets (in Kisoro) and farm-gates (both districts) were either slaughtered at designated slaughter slabs or within pork joint premises. Consumers in both districts could access fresh pork from pork-kiosks (butcheries) and pork-joints. In both districts, farmers acquired breeding stock from fellow farmers.

### Focus group discussions

According to the participants in both farmers and traders’ FGDs, the ban on trade and movement of pigs and pig products and pork consumption were the imposed quarantine measures. Several actions were taken by the stakeholders in response to the imposed quarantine measures (see Table 3). These actions and the corresponding measures are described for each of the actors in the subsequent sections.

### Farmers

Fourteen different actions were mentioned in the farmer FGDs in response to the restrictions on trade and movement of pigs and pig products. Participants in three of the four farmer FGDs reported that farmers did not buy pigs (e.g,. breeding stock) during the restrictions period. Continuous sales of pigs to traders were however mentioned in all the farmer FGDs. Participants in one of the four farmer FGDs (FGD1) reported to offer traders to slaughter pigs at their homesteads during the quarantine period. It was further mentioned that pigs were transported from farmers’ home using boda-boda.^2^

### Traders

Twenty-four different actions were mentioned in the trader FGDs in response to the quarantine restriction. Participants mentioned that during the quarantine period some traders avoided pig transactions in livestock markets and instead performed pig trade at farmers’ homesteads. Participants further reported having sold pigs and pig products both within the two districts and beyond district and country borders. Both live pigs and pig products were transported from farmers’ homesteads to clients using boda-boda. Participants reported that no pigs were slaughtered in slaughter slabs, and all the pork joints were un-operational during the quarantine period. Several participants mentioned that farmers’ homesteads were used for slaughtering pigs instead of slaughter slabs. Traders further reported that consumers were buying pork from some traders despite closure of slaughter slabs, butcheries and pork joints.

### Veterinarians

Six different actions taken to enforce the ban on pig trade and movement were mentioned in the veterinarian FGDs. Participants reported that pig trading was not carried out in any of the livestock markets and movement permits were not issued to pig traders during the quarantine period. They further mentioned involvement of crime preventers^3^ in the enforcement of restrictions in livestock markets to ensure that no pigs accessed the markets. Participants said that they had conducted surveillance visits in sub-counties with ASF outbreaks to check if traders closed their business premises (such as slaughter-slabs and pork joints) as obliged, and to get a better understanding of the epidemiological situation.

### Perceived impact of quarantine measures on stakeholders

#### Farmers

Overall, farmers were perceived to be the most affected stakeholder; ranked first by three out of four farmer FGDs, two out of four trader FGDs, and both veterinarians FGDs (see Table 4). Impacts mentioned in all FGDs included revenue foregone when marketable pigs could not be sold and the additional cost of feeding marketable pigs during the quarantine period. Additionally, costs associated with constructing temporary pig shelters, feeding of the pigs that would have been managed on free-range if restrictions had not been in place, and the opportunity cost foregone of selling pig manure were mentioned by participants in the four farmers’ FGDs.

#### Butchers and pork joint operators (pork retailers)

Pork retailers were perceived to be the second most affected stakeholder, being ranked either in first or second position by three out of four farmer FGDs, all four trader FGDs and one of two veterinarian FGDs. According to participants across all FGDs, failure to acquire pigs from farmers as well as loss of income from pigs and pork sales was perceived as negatively affecting business of butchers and pork joint operators when markets, pork butcheries and pork joints were closed. Losses due to advance rental payment for business premises, and cost of relocating pork-roasting businesses to neighbouring districts were mentioned by all four traders FGDs (see Table 5). A participant in FGD2 exemplified this; “Big, big loss by pork roasters as they never attempted to roast in fear of people detecting smell and smoke from pork joints.”

#### Middlemen

The overall economic impact of quarantine on middlemen was reported as small compared to farmers and pork retailers. Participants in nine FGDs perceived that middleman had lost income from sale of pigs and commission for mediating pig sale transactions between farmers and butchers during the restriction period (see Table 5). Participants noted that middlemen normally make their business from buying pigs from farmers and selling to butchers, or payments are received from both farmers and butchers for linking transactions. Two traders and one veterinarians’ FGDs perceived that middlemen were affected as money that were paid in advance for renting pig holding facilities that remained non-operational during the restriction period could not be recuperated.

#### Tax-collectors

Sellers and buyers of pigs pay market-dues and loading fees in livestock markets. Five FGDs (two farmers’, two traders’, and one veterinarians’ FGDs) mentioned that the collectors of these fees at the livestock markets were affected by the imposed quarantine. The perceived loss of pig slaughter-fees were reported by six FGDs (all four traders’ s and all two veterinarians’ FGDs) to have affected tax-collectors when slaughter slabs and pork joints were closed. It was reported that even when the quarantine was lifted, tax-collectors were not reinstated to complete their task.

#### Consumers

Consumers and veterinarians were considered the least affected stakeholders, ranked in either fifth or sixth positions by three out of four farmer FGDs, all four trader FGDs and both veterinarian FGDs. All FGDs reported that consumers incurred additional cost for buying substitute for pork that was more expensive (e.g., fish and chicken), and six (four farmers and two traders’ FGDs) mentioned costs for hiring boda-boda riders to search for pork*.* The price of pork bought illegally was perceived to be higher than the normal pork price by two farmers and two traders FGDs.

#### Veterinarians

Three traders’ and both veterinarians’ FGDs mentioned that veterinarians lost fees for pork inspection, pork quality assurance at slaughter slabs and movement permit for health certification of pig on transit. The foregone cost of inspection-fee for ante-mortem examination of live pigs in livestock markets was perceived as affecting veterinarians by two traders and one veterinarian FGDs. Costs of fuel for collecting pig carcasses from farmers and burning carcasses at district head quarter incurred by veterinarians was mentioned by two farmers and one veterinarian FGDs.

## Discussions

The study assessed actions different stakeholders took and the perceived economic impact during quarantine imposition. While the restriction measures seemed to be largely observed in the formal market spaces, a concurrent shift from formal market settings to more informal, non-regulated settings were described, suggesting that the regulatory authorities only managed to enforce quarantine in the formal marketplaces. Continuous trade in live pigs and pig products has previously been reported as common during ASF outbreak and subsequent quarantine in Uganda (Chenais et al. 2017b; Dione et al. 2017; Aliro et al. 2022; Okello et al. 2022). Another study pointed to lack of capacity to enforce existing regulation as a key constraint in implementing ASF quarantine (Wesonga et al. 2018).

With trade moving from formal to informal markets, stakeholders do not pay any fees (mandatory movement permit fees, slaughter fees and carcasses quality certification fees are performed by the public veterinary personnel as legally stipulated in the Public Health Act (Laws of Uganda 1935), Cattle Traders Act (Laws of Uganda, 2000) and the Animal Diseases Act (Laws of Uganda 2006)), and these incomes were reported as foregone for the tax collectors. In Uganda, the fees generated from mandatory certification are used to sustain the continuity these activities and form part of the salary for the public veterinarians as the allocated budget often are inadequate (Ilukor 2018). During the quarantine the role of the regulatory authorities thus changes from facilitation of movement and providing certification to inhibiting the same.

Animal disease control measures associated with negative economic impact and that is not accompanied by compensation is bound to non-compliance (OIE 2014). In Uganda, farmers are not compensated for loss of pigs due to ASF outbreaks, reporting suspected outbreaks are thus mostly non-beneficial for farmers. Due to denying farmers the opportunity to sell livestock and the lack of benefit offered to them, the implementation of quarantine is likewise unpopular among other stakeholders including political leaders who can lift quarantine as incentives to appease their voters (Ilukor et al. 2015).

Many smallholders in east Africa rely on pigs as the main source of revenue, with pig sales financing critical household needs such as school fees and healthcare (Mutua et al. 2011; Muhanguzi et al. 2012). Poor people such as most smallholders in the study area are more vulnerable to external disturbances such as quarantine following animal disease outbreak (Wagstaff 2006), are highly dependent on pigs, and this neccessitates prioritising the continuation of this business activity also during quarantine. While the impacts associated with livestock disease outbreaks and control affect actors all along the value chain in some ways (Rich & Wanyoike 2010)*,* the impact is not the same for all stakeholders, depending among other things on livestock’s contribution to their livelihood (Rich & Perry 2011). In this study economic impact of quarantine was perceived to be higher among farmers and pork retailers. According to the results, traders seemed to be less affected by quarantine than farmers. Traders relocate their business to areas without quarantine. Previous studies have further reported of traders making profit during ASF outbreaks by buying pigs cheap and selling pork at normal market price (Chenais et al. 2017b). This is exacerbated as smallholders lack a common voice in pig trade (Ouma et al. 2016).

In Uganda, over 70% of pork is consumed at pork joints (Roesel et al. 2019). Consequently, quarantine restrictions were perceived as causing revenue losses affecting pork retailers. In this study, the continued trade and slaughter of pigs however implied consistent consumer demand.

This study had some limitations that must be pointed out since they could affect the interpretation and application of the findings. First, there was an unbalanced gender distribution among the participants. Although men often decide on how to allocate resources for biosecurity, women play a key role in pig management (Ouma et al. 2015) and have substantial autonomy on daily expenses, and high bargaining power for family business (Agarwal 1997). Thus, the selection bias with underrepresentation of women could have affected the results. Second, the study was conducted in 2019 but concerned quarantine that was imposed between 2015 and 2018. Consequently, recall bias might have been present (Raphael 1987). To avoid this, future studies could be conducted immediately after a quarantine to allow for close-to-real time assessment of economic impact of the quarantine. Lastly, loss of information or depth could have occurred as the FGDs were conducted with the help of translators (Rufumbira in Kisoro district; and Madi in Moyo district) (Fischer et al. 2020). However, selecting bi-lingual facilitators that were resident veterinarians in the districts, with broad experience in activities along pig value chain, and triangulating data with the facilitators post-interview helped to overcome this potential bias.

In conclusion, mandatory quarantine regulations seemed to have been implemented in formal market places, but trade continuing in informal marketplaces. The movement of live pigs and pig products continued, with pork consumed at home. The results suggest that not only the disease but also its control had negative impact on all the stakeholders although different stakeholders were perceived to be differently affected by the quarantine, with farmers perceived as most affected. The impact of the control was not quantified in this study, neither was the cost–benefit of the quarantine in terms of controlling the disease. These areas could all be explored in the future.

### Supplementary Information

Below is the link to the electronic supplementary material.

Supplementary file1 (DOCX 16 KB)

## Data Availability

The data associated with the manuscript are available from the corresponding author upon reasonable request.

## Footnotes

1 “Pork-joints” are small kiosk-like restaurants serving only pork.

2 “Boda-Boda” is a term in East-Africa for motorcycle used as a taxi for carrying a passenger and/or goods.

3 “Crime preventers” are a volunteer force of civilians recruited and managed by police to report on and prevent crime in cooperation with the police and communities, in Uganda.
