# Supplementary material for: Actions and perceived impact of African swine fever control measures along the smallholder pig value chain in Uganda
Source: Trop Anim Health Prod. 2023 Nov 21;55(6):410. doi: 10.1007/s11250-023-03828-5 (PMC10663180; doi:10.1007/s11250-023-03828-5)
Supplement: Supplementary file 1 — Supplementary file1 (DOCX 16 KB) [file 11250_2023_3828_MOESM1_ESM.docx]

**ANNEX**

**ANNEX 01: KEY INFORMANT INTERVIEW (KII) GUIDES**

**Target:** **District Veterinary Officers (DVO)**

This courtesy call on your office is in regards to quarantine imposed here in the past. We are conducting a study on implementation and economic impact of past quarantine along pig value chains in Kisoro and Moyo districts. The findings and recommendations from this academic study will guide the Government on future actions to improve pigs and pork movement restrictions during African swine fever outbreaks. Based on this back ground, I will interview you using these few questions;

**Question one:**

Generally, describe the marketing dynamics of pigs and pork in your district. Mention, if any the available pig livestock markets, and sourcing for breeding stocks.

**Question two:**

How would you describe logically the pig value chain in the district, from farmers through different market linkages to the final pork consumer at the time of pig and pork movement restrictions?

**ANNEX 02: FOCUS GROUP DISCUSSION (FGD) GUIDES**

Targeted Pig Value Chain Actor: **Farmers/Traders/Veterinarians**

Date__________________Starting time___________Finishing time____________________

Subcounty represented________________________________________________________

Total number of participants in the group:_________________________________________

Facilitator_______________________________Note-taker___________________________

Objective

To document the information needed to design a questionnaire on the possible impacts of ASF quarantine.

Methodology

Focus group discussions

Material

Flip chart, digital camera, counters (bean seeds), markers, manila paper, masking tape, rulers, sodas and water, biscuits

**Introductory section: by facilitator**

1. Greet the participants and request one member to open with a word of prayer.
2. Self-introduction on both sides (participants and team members).
3. Setting the scene: Introduce the project, highlighting the objectives and the important role of the participants in meeting the objectives.
4. Take them through the planned process of the focus group discussions.
5. Take them through the consent form
6. Ask for consent to use tape recorders and cameras (if any).
7. Set the ground rules together with them.

**A. Identification of ASF - quarantine control measures**

1. Which ASF control measures were imposed by the Government authorities to implement the quarantine? (List the control measures on flip chart).
2. Why was the control measure imposed?
3. What actions/measures did you take following imposition of control measures?

**B. Experience on the Impact of the latest ASF quarantine**

1. Who were the affected pig Stakeholders? (listing)
2. Rank the effects of ASF on the listed stakeholders in the order of relative monetary losses and give reasons for your decisions. [Probe into detail cost implication (cost incurred and cost forgone) in relation to; implementation of the imposed control measures; live pigs and pork]
3. Give reasons for your decisions on the ranking in #2
